# Rhesus Macaque Activating Killer Immunoglobulin-Like Receptors Associate With Fc Receptor Gamma (FCER1G) and Not With DAP12 Adaptor Proteins Resulting in Stabilized Expression and Enabling Signal Transduction

**DOI:** 10.3389/fimmu.2021.678964

**Published:** 2021-04-23

**Authors:** Mohammad Zahidul Hasan, Lutz Walter

**Affiliations:** Primate Genetics Laboratory, German Primate Center, Leibniz Institute for Primate Research, Göttingen, Germany

**Keywords:** adaptor association, Co-immunoprecipitation (co-IP), rhesus macaque (Macaca mulatta), DAP12, FCER1G, activating KIR, Activating killer cell immunoglobulin-like receptors, NK cell

## Abstract

Activating killer cell immunoglobulin-like receptors (KIR) in macaques are thought to be derived by genetic recombination of the region encoding the transmembrane and intracellular part of KIR2DL4 and a KIR3D gene. As a result, all macaque activating KIR possess a positively charged arginine residue in the transmembrane region. As human KIR2DL4 associates with the FCER1G (also called Fc receptor-gamma, FcRγ) adaptor, we hypothesized that in contrast to human and great ape the activating KIRs of macaques associate with FcRγ instead of DAP12. By applying co-immunoprecipitation of transfected as well as primary cells, we demonstrate that rhesus macaque KIR3DS05 indeed associates with FcRγ and not with DAP12. This association with FcRγ results in increased and substantially stabilized surface expression of KIR3DS05. In addition, we demonstrate that binding of specific ligands of KIR3DS05, Mamu-A1*001 and A1*011, resulted in signal transduction in the presence of FcRγ in contrast to DAP12.

## Introduction

Stimulatory NK cell receptors usually do not transmit signals directly but through association with activation motif-containing accessory proteins DAP12 and FcRγ (also called γ and encoded by the FCER1G gene). This non-covalent interaction is mediated by basic residues (lysine and arginine) in the transmembrane region of the receptor and an acidic residue (aspartic acid) in the adaptor proteins. The prototypical activating KIRs in human interact with DAP12 (1–4), while KIR2DL4 is the only KIR known so far to associate with the FcRγ adaptor (5). Responsible for this different usage of adaptor proteins are positions of the charged amino acid residues in the transmembrane regions (6): a lysine and an aspartic acid residue can be found in prototypical KIRs and DAP12 at position 9, respectively, while KIR2DL4 has an arginine and FcRγ an aspartic acid residue at position 4 and 3, respectively. Both adaptors contain a single immunoreceptor tyrosine-based activation motif (ITAM) of the sequence D/ExxYxxL/I(x_6–8_)YxxL/I in their cytoplasmic region, which is responsible for signal transduction upon binding of a specific ligand by the associated receptor. In addition, these adaptor proteins contribute to stabilization of the cell surface expression of interacting stimulatory receptors of different types such as CD16 (7), NKp30 and NKp46 (8), NKp44 (9), KIR (4, 5, 10), CD94/NKG2C (3, 11), ILT1 (12) and many others (13).

KIR genomics in rhesus macaques is more complex as in humans (14–21) and the genomic diversity and plasticity are focused on lineage II KIR, i.e. to genes encoding three-domain KIR proteins. In contrast to the other *KIR* genes, human and macaque *KIR2DL4* are orthologous (14, 22). Interestingly, the activating rhesus macaque *KIR* genes were formed by recombination between a *KIR3D* gene and *KIR2DL4* (14) and subsequent splice site mutation in intron 8 (23). Thus, the transmembrane region of activating KIR proteins and of KIR2DL4 are very similar. Hence, all activating rhesus macaque KIR proteins possess an arginine residue at position 4 of the transmembrane region instead of a lysine residue that is found in human and great ape activating KIR at position 9. Which of the two adaptor proteins can associate with macaque activating KIR was so far unknown. Here we demonstrate that a prototypical rhesus macaque activating KIR protein indeed interacts with FcRγ and not with DAP12.

## Materials and Methods

### Rhesus Macaque PBMC Samples

Peripheral blood samples of 8 rhesus macaques kept at the German Primate Center were obtained from the Animal Husbandry Unit of the DPZ for MHC class I genotyping purposes. Peripheral blood mononuclear cells (PBMC) were isolated as described previously (24). Briefly, peripheral blood was diluted with RPMI medium (1:1) and transferred to a leucosep tube filled with ficoll. After 40 min centrifugation at 800 x g, the PBMC layer was removed carefully and the cells were washed with RPMI. Leftovers of PBMCs from genotyping were used for co-immunoprecipitation experiments. As the exact *KIR* genotype of the animals was not known, we pooled PBMCs of 4 animals (about 5x10^6^ cells in total). Two pools of samples were frozen and stored at -140°C until use for co-immunoprecipitation (see below).

### Expression Constructs

The rhesus macaque KIR3DS05 with a C-terminal AcGFP tag was described before (25). We cloned C-terminal myc-tagged versions of either rhesus macaque DAP12 or FcRγ in multiple cloning site A of the pIRES expression plasmid (Clontech) and AcGFP-tagged KIR3DS05 in multiple cloning site B. Multiple cloning sites A and B in this vector are separated by an internal ribosomal entry site (IRES). Such bicistronic mRNA expression ensures simultaneous expression of the activating KIR3DS05 and either the DAP12 or the FcRγ adaptor protein in transfected cells.

### Transfection and Transfected Cell Lines

Plasmid DNA was transfected at 1:3 ratio using Lipofectamine 2000 (Thermo Fisher Scientific) into overnight exponentially grown HEK-293 or HeLa cells in 6-well plates. Transfected cells were cultured in the Medium_HEK293_ (DMEM, 10% inactivated FBS and 0.1% Gentamycin) and incubated for ˜ 60 – 65 hours at 37°C with 5% CO_2_. Stably transfected HEK-293 cells were obtained by selection in neomycin-containing medium for at least 14 d. KIR3DS05_AcGFP_, KIR3DS05_AcGFP_+FcRγ_myc_, or KIR3DS05_AcGFP_+DAP12_myc_ in pIRES plasmid DNA were also transfected in Jurkat cells using the electroporation-based transfection system Nucleofector II according to the supplier’s (Lonza) information for Jurkat cells and these cells were cultured in Medium_Jurkat_ (RPMI, 10% inactivated FBS and 0.1% Gentamycin) at 37°C with 5% CO_2_.

### Flow Cytometry and Cell Sorting

Transfected HEK-293 or HeLa cells (1 – 2 × 10^6^) were harvested using warm (37 °C) 1x DPBS, centrifuged (5 min, 200 × g) and resuspended in 100 μl staining solution_A_ (1x PBS, 0.5% BSA and 0.05% sodium azide) in 1.5 ml tubes. For staining of KIR3DS05, 1.1 μg of monoclonal mouse anti-macaque pan-KIR3D antibody 1C7 (26) was applied for 1 hour at RT. After the incubation, cells were washed with 1xDPBS and 1 μl APC goat anti-mouse-IgG antibody (Biolegend) was used in resuspended cells in 100 μl staining solution_A_ for 30 min at RT.

For intracellular staining of myc-tagged adaptor proteins, cells were fixed with fixation buffer (Biolegend), permeabilized with intracellular staining permeabilization wash (Biolegend), and blocked with blocking buffer (1x PBS and 2% BSA). Rabbit-anti-myc-tag antibody (1:150) (Cell signaling technology) was then applied for 40 min at RT. Finally, 1 µl Brilliant Violet 421 conjugated donkey anti-rabbit IgG secondary antibody (Biolegend) was added and incubated for another 30 min at RT with staining solution_A_.

Live/dead staining of cells was performed in 100 μl ice cold Zombie aqua fixable viability kit (Biolegend) for 10 min at RT (dark) and dead cells were excluded from analysis. Cells were analyzed in a BD LSR II flow cytometer (BD Biosciences) and data were analyzed using FlowJo 10.7.

Cells were sorted using the SH800 cell sorter (Sony) and subsequently cultured in appropriate culture medium.

### Analysis of Stabilization of KIR3DS05 Expression by Adaptor Proteins

After antibiotic selection, stably expressing KIR3DS05_AcGFP_, KIR3DS05_AcGFP_+FcRγ_myc_ and KIR3DS05_AcGFP_+DAP12_myc_ HEK-293 cells were suspended in sorting buffer (1x PBS, 2% FCS and 2 mM EDTA). Only AcGFP-expressing cells were then gated and equal number of cells (around 80 – 90% AcGFP-positive) were sorted and cultured in Medium_HEK_. We then reanalyzed the cells using antibody 1C7 and used identical frequency of 80 – 90% KIR3DS05-positive cells as starting point in all experiments. After different passages with intervals of two to three days, an aliquot of cells was removed after each passage to measure KIR3DS05 expression by flow cytometry.

### Stimulation of KIR3DS05 and Adaptor Protein-Expressing Cells

Stimulation of KIR3DS05 was performed using HEK-293 cells expressing AcGFP-tagged MHC class I ligands Mamu-A1*001 or A1*011 and as control the non-interacting Mamu-B*030 (25). Jurkat cells expressing KIR3DS05 with and without adaptor proteins (see above expression constructs) were incubated with these Mamu class I-expressing HEK-293 cells (overnight exponentially grown) in 12-well plates for 14 hours in Medium_Jurkat_. As readout of stimulation *via* KIR3DS05 and associated adaptor protein, we measured expression of CD69 by flow cytometry. The non-adherent Jurkat cells were carefully removed from the adherent HEK-293 cells, centrifuged (5 min, 300 x g) and resuspended in 100 μl staining solution_A_ for staining with 3 μl APC anti-human CD69 antibody (Biolegend) for 40 min at RT. Jurkat cells were differentiated from accidentally transferred HEK-293 cells in flow cytometry based on forward and side scatter characteristics.

### Co-Immunoprecipitation (Co-IP)

About 1 – 2 × 10^6^ sorted HEK-293 cells stably expressing KIR3DS05_AcGFP_, KIR3DS05_AcGFP_+FcRγ_myc_ or KIR3DS05_AcGFP_+DAP12_myc_ were grown individually in T25 tissue culture flask in Medium_HEK293_ at 37°C with 5% CO_2_. The culture medium was discarded and the cells were washed with ice cold 1× DPBS. After discarding the DPBS, cells were lysed in 400 μl ice cold lysis buffer (10 mM Tris-HCl pH 7.5, 150 mM NaCl, 1% N-Dodecyl β-D-maltoside (Thermo Fisher Scientific), 0.4 mM EDTA and 1 tablet protease-inhibitor-cocktail complete mini (Roche Diagnostic)) for 20 min at 4°C with gentle shaking. After centrifugation (15 min, 16000 × g), the collected lysate was pre-cleared using 40 µl Protein G sepharose beads (GE healthcare) and the protein concentration of the lysate was determined in a Qubit 4 fluorometer using Qubit protein assay kits (Thermo Fisher Scientific). Subsequently, 4.4 μg of monoclonal antibody 1C7 was added to ˜40 μg pre-cleared lysate and incubated overnight at 4°C with rotation. Protein G beads were added and incubated for another 5 hours at 4°C. After washing the beads, bound proteins were released by incubation in 30 μl 5x SDS-PAGE reducing protein loading buffer (Bosterbio) at 95°C for 5 min and centrifuged for 5 min, 10000 × g at RT. The supernatant was collected and either used directly for SDS gel electrophoresis or was stored at -20°C.

About 5 × 10^6^ rhesus macaque pooled PBMCs were lysed as described above and pre-cleared lysates were subjected to Co-IP with 1C7 antibody in reducing buffer. The collected samples were ready to use for SDS-page or stored at – 20°C.

### Western Blot

Prepared lysate and co-immunoprecipitated protein samples (˜ 16 μl) were separated in 14% Bis-Tris gels or 8 – 16% Mini-protean-TGX stain free protein gels (Bio-Rad) and blotted on a nitrocellulose pure transfer membrane (Ultracruze) or Trans-Blot Turbo mini PVDF (Bio-Rad) using the Trans-Blot Turbo Transfer system (Bio-Rad). After blocking in Pierce protein-free blocking buffer (Thermo fisher scientific), FcRγ_myc_ or DAP12_myc_ were detected using 1 μg/ml rabbit anti-myc antibody (1:1000; Abcam) and a secondary antibody goat anti-rabbit immunoglobulin horse radish peroxidase (HRP)-conjugated (1:1500; Dako) in reducing condition in blocking buffer. FcRγ and DAP12 were detected in rhesus macaque PBMCs using polyclonal antibodies goat anti-FcRγ and rabbit anti-DAP12 (both with 1:1000 dilution), and as secondary antibodies HRP-conjugated rabbit anti-goat immunoglobulin and goat anti-rabbit immunoglobulin (both in 1:1500 dilution; all polyclonal antibodies from Santa Cruz Biotechnology) in Every blot blocking buffer (Bio-Rad).

### Confocal Laser Microscopy

Preparation and staining of transiently transfected HEK-293 cells were the same as described above for flow cytometry. Cells were stained with 1C7 antibody and as secondary antibody 1 μl of PE goat anti-mouse IgG (Biolegend) to detect cell surface-expressed KIR3DS05 and were subsequently fixed, permeabilized, blocked and stained intracellularly with 0.5 μg anti-myc antibody (Abcam) and 0.25 μg APC conjugated goat anti-rabbit IgG (Abcam) to detect myc-tagged adaptors. Finally, a drop of Fluoromount-G mounting medium with DAPI (Thermo Fisher Scientific) was applied on a microscope glass slide (Carl Roth), and the stained cells were transferred onto the mounting medium and incubated for 5 min at RT, before fixing the cells with a cover slip (24 × 24 mm). The images were captured with the Plan-Apochromat 63x/1.40 oil objective in confocal laser microscope LSM 800 (Carl Zeiss) fitted with ZEN 2.3 software (Carl Zeiss) and mean fluorescence intensity (MFI) value of KIR3DS05 (1C7) and FcRγ/DAP12 (anti-myc) in 11 individual stained cells were measured using the same software.

### Statistical Analysis

Differences between two groups were analyzed by applying a Student’s t-test (parametric, unpaired, two-tailed, 95% confidence level) using GraphPad Prism 9. Differences between groups with p values >0.05 were regarded as not statistically significant.

## Results

### Identification of the Adaptor Protein for Rhesus Macaque Activating KIR

**Figure 1:**
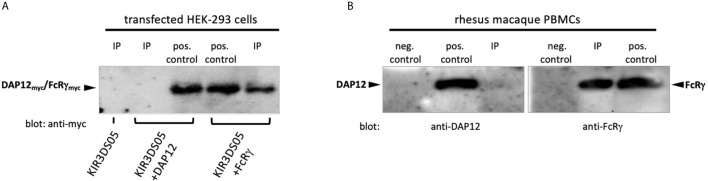
Co-immunoprecipitation of adaptor proteins. **(A)** Immunoprecipitation of KIR3DS05 was performed with anti-rhesus macaque pan-KIR3D antibody 1C7 (26) as indicated (IP). As positive control we used cell lysates of KIR and adaptor protein-expressing cells. Anti-myc antibody was used in blots to detect myc-tagged FcRγ and DAP12 adaptor proteins. A representative figure is shown from five independent experiments. The molecular weight of rhesus macaque FcRγ and DAP12 is 13.2 and 10.8 kDa, respectively, and the size difference is hardly detectable in the western blot analyses. **(B)** Lysates of rhesus macaque PBMCs were used directly (pos. control) or were subjected to immunoprecipitation with antibody 1C7 (IP). Detection of adaptor proteins in western blots was performed with anti-FcRγ and anti-DAP12 polyclonal antibodies. To control for unspecific binding of the polyclonal anti-adapter protein antibodies in western blot, we used FcRγ and DAP12 negative cells (untransfected HEK-293 cells; neg. control). The results from one out of two independent experiments are shown.

To find out the interacting adaptor proteins of activating KIR proteins in rhesus macaques, we expressed AcGFP-tagged KIR3DS05 alone or together with myc-tagged FcRγ or DAP12 adaptor protein in HEK-293 cells. Equal numbers of KIR3DS05_AcGFP_ expressing cells were sorted and their lysate was used for Co-IP with anti-rhesus macaque KIR antibody 1C7 (26). The cell lysis buffer contained N-Dodecyl β-D-maltoside, which was previously shown to be superior to other detergents in identifying receptor-adaptor complexes in NK cells (9). Western blot analysis with an HRP-conjugated anti-myc antibody was performed to detect which adaptor protein is associated with KIR3DS05. Bands of the expected size of 10 –13 kDa were detected for both FcRγ and DAP12 in the respective control samples (**Figure 1A**). When KIR3DS05 was immunoprecipitated, we detected only the FcRγ protein, but not DAP12 (**Figure 1A**). To confirm this finding obtained from transfected cells and tagged adaptor proteins, we immunoprecipitated KIR proteins from two pools of rhesus macaque PBMCs with anti-rhesus pan-KIR3D antibody 1C7 (26) and used polyclonal antibodies against FcRγ and DAP12 in western blots. In accord with the transfection experiments, also in primary cells only FcRγ and not DAP12 was found in immunoprecipitated PBMC samples (**Figure 1B**). These findings clearly demonstrate that stimulatory KIR in rhesus macaques interact with the FcRγ adaptor protein and not with DAP12.

### FcRγ Adaptor Protein Promotes High Cell Surface Expression of Rhesus Macaque Activating KIR

**Figure 2:**
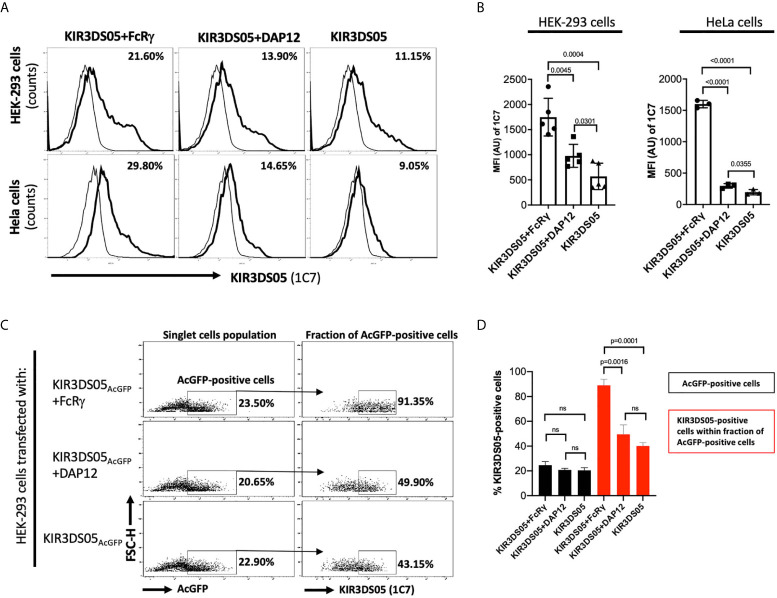
Flow cytometry analysis of rhesus macaque KIR3DS05 cell surface expression in the presence of myc-tagged FcRγ or DAP12 adaptor proteins. **(A)** Histograms of a representative experiment of HEK-293 and HeLa cells transiently transfected with AcGFP-tagged KIR3DS05, either alone or in combination with either DAP12 or FcRγ (thick lines). Monoclonal anti-rhesus macaque KIR antibody 1C7 was used to detect KIR3DS05 expression indicated as percent positive cells. Untransfected HEK-293 and HeLa cells were used as respective negative controls of 1C7 staining (thin lines). **(B)** Mean fluorescence intensity of KIR3DS05 expression in transfected cells among independently performed experiments (HEK-293 n=5; HeLa n=3). **(C)** HEK-293 cells after transfection with AcGFP-tagged KIR3DS05 were gated on the singlet cells and then 1C7-stained cells were measured within the fraction of AcGFP-positive cells population. A representative experiment is shown. **(D)** 1C7-stained cells within the AcGFP-positive fraction were measured in individual experiments (n=3). ns, statistically not significant.

Enhanced cell surface expression was previously demonstrated for human KIR2DS1, KIR2DS2, KIR2DS4 (10), and KIR3DS1 (4) in the presence of DAP12, and for human KIR2DL4 in the presence of FcRγ (5). Thus, we transiently transfected AcGFP-tagged KIR3DS05 with or without adaptor proteins in HEK-293 and HeLa cells and compared its cell surface expression in flow cytometry. In both cell lines, KIR3DS05_AcGFP_ revealed about 2 – 3 times higher cell surface expression in the presence of FcRγ as compared to the presence of DAP12 or without any adaptor (**Figures 2A, B** **)**. The enhanced expression is seen in both %-positive cells and mean fluorescent intensity. This higher expression is not due to transfection efficiency as reflected by comparable GFP expression in both experimental settings (**Figure 2C**), while 89.0% ( ± 4.8%) of these GFP-positive cells express KIR3DS05 in combination with FcRγ and only 40.2% (± 2.7%) of the GFP-positive cells express KIR3DS05 in the absence of any adaptor protein or 49.6% ( ± 7.6%) with DAP12 **(** **Figures 2C, D** **)**.

**Figure 3:**
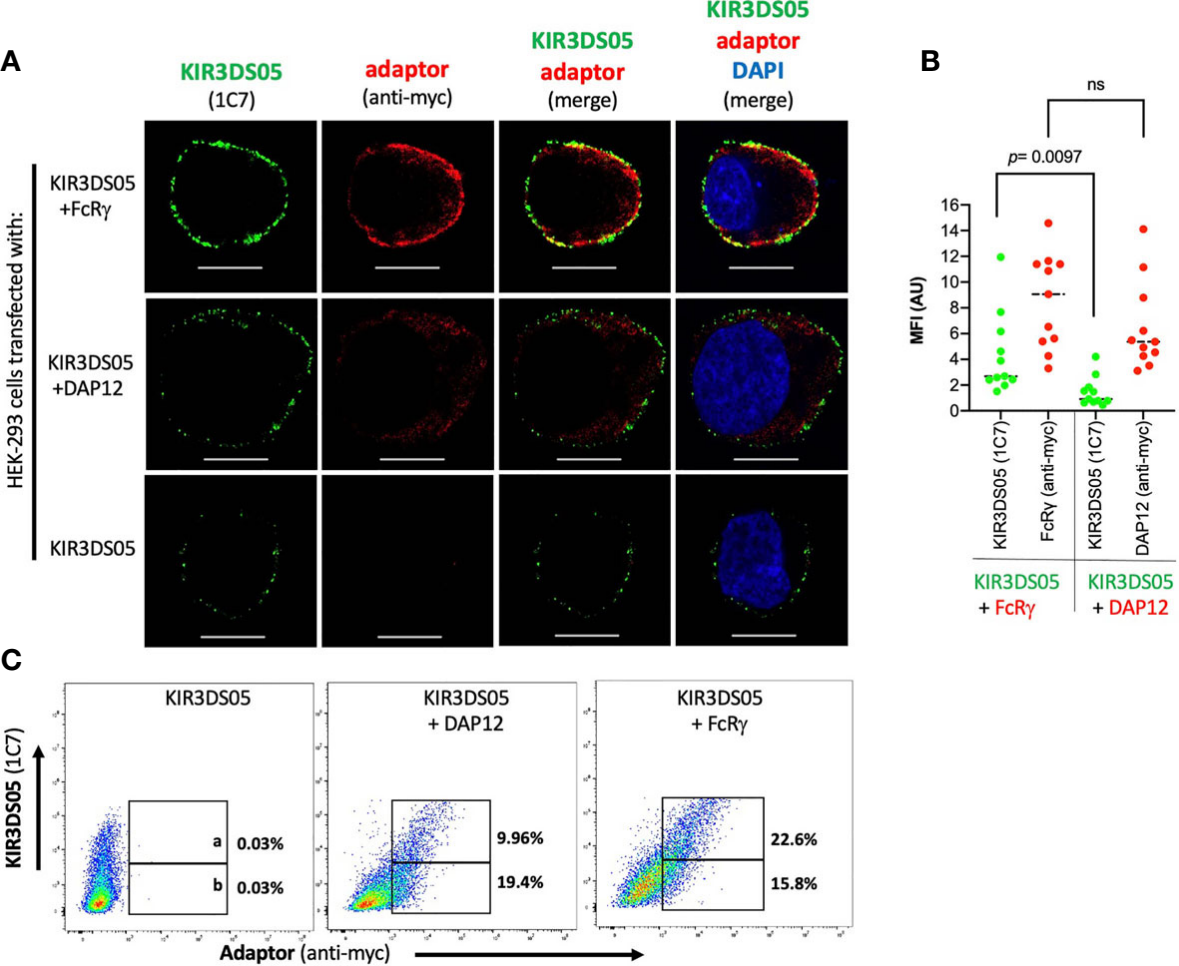
Confocal laser microscopy analysis of rhesus macaque AcGFP-tagged KIR3DS05 cell surface expression in the presence of myc-tagged FcRγ or DAP12 adaptor proteins. **(A)** Cells were stained as indicated. In the merged figures, yellow color indicates co-localization of KIR3DS05 (green) and adaptor (red). DAPI staining (blue) shows cell nuclei. Scale bar represents 10.20 µm. **(B)** Single confocal sections of 11 randomly chosen individual cells were also used to analyze mean fluorescence intensity values using the ZEN software (version 2.3). The means of columns were calculated and corresponding experiments were compared. Statistical significance of differences is shown. ns, not significant. **(C)** Flow cytometric analysis of correlation of cell surface expressed KIR3DS05 in combination with adaptor proteins in cells stained with 1C7 and anti-myc antibodies. The gates indicate: a = 1C7 and myc-positive; b = only myc-positive.

Expression of KIR3DS05_AcGFP_ with myc-tagged FcRγ and DAP12 in transfected HEK-293 cells was also investigated by confocal laser microscopy. Strong signals of KIR3DS05 (green) and co-localization of KIR3DS05 with FcRγ_myc_ (red) complexes were detected, whereas weaker signals of KIR3DS05 and no clear signs of co-localization with DAP12_myc_ were observed (**Figure 3A**). The measured MFI from a comparable area among these cells shows stronger KIR3DS05_AcGFP_ expression when FcRγ_myc_ is present as compared to co-expression with DAP12 (**Figure 3B**). The positive correlation of KIR3DS05 and myc expression was more evident with FcRγ (**Figure 3C**). The ratio of % cells expressing KIR3DS05 and adaptor and only the adaptor being 1.4 in the combination of KIR3DS05 with FcRγ and 0.5 in combination with DAP12.

As KIR3DS05_AcGFP_ was expressed from a bicistronic mRNA with either FcRγ_myc_ or DAP12_myc_, the similar amount of the two adaptor proteins in both settings implies that also comparable amounts of KIR3DS05 protein are present. Thus, the observed differences in KIR3DS05 cell surface expression are due to functional differences in pairing of KIR3DS05 with these two adaptor proteins and not to experimental variation.

### FcRγ Adaptor Protein Stabilizes Cell Surface Expression of Rhesus Macaque KIR3DS05

**Figure 4:**
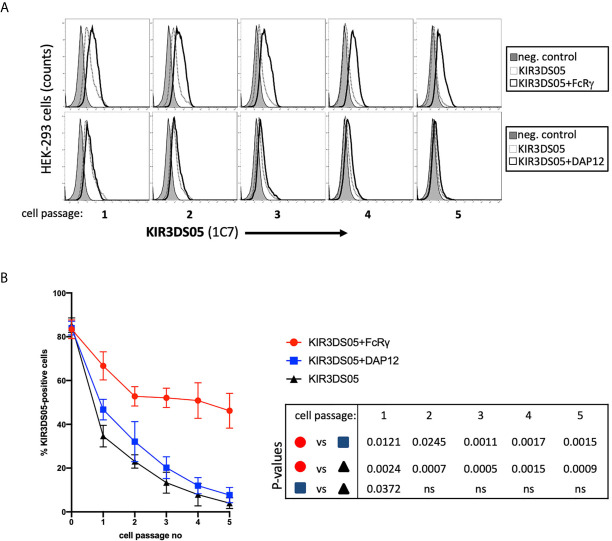
Stability of AcGFP-tagged KIR3DS05 expression in the presence of myc-tagged FcRγ and DAP12 adaptor proteins. HEK-293 cells stably transfected with AcGFP-tagged KIR3DS05 either alone or in combination with either myc-tagged FcRγ or DAP12 adaptor proteins were followed for KIR3DS05 expression over time (one cell passage corresponds to 2-3 days). **(A)** Flow cytometric analysis of KIR3DS05 cell surface expression measured with antibody 1C7 after each passage of the cells. **(B)** Identical percentages of KIR3DS05-positive proteins were used as starting point (passage 0) and expression of KIR3DS05 (% positive cells) was measured after each passage of the cells. The results of three independent experiments and of unpaired t tests are shown (ns, not significant).

As the proper adaptor protein stabilizes expression of activating human KIR proteins, we tested whether the presence of FcRγ also results in stabilized KIR3DS05 cell surface expression. Thus, we sorted 80-90% AcGFP-positive HEK-293 cells stably transfected with either KIR3DS05_AcGFP_, KIR3DS05_AcGFP_+FcRγ or KIR3DS05_AcGFP_+DAP12 and stained these cells with 1C7 antibody to measure KIR3DS05 cell surface expression in the different cell passages every 2 - 3 days (**Figure 4A**). KIR3DS05 expressed in the absence of any adaptor protein rapidly leads to reduced cell surface expression over time and a similar loss was noticed for KIR3DS05 in the presence of tagged DAP12: only about 3 - 7% KIR3DS05-positive cells remained after 5 passages (**Figure 4B** **).** Contrasting this loss is a relatively stable expression of KIR3DS05 in the presence of FcRγ and only a slow reduction noticed over time (**Figures 4A, B** **)**. The differences between KIR3DS05_AcGFP_+FcRγ and the other two conditions are statistically significant for all time points, whereas the comparison between KIR3DS05_AcGFP_+DAP12 and KIR3DS05_AcGFP_ is statistically significant only after passage 1 (p=0.0372) but not thereafter (**Figure 4B**). Thus, FcRγ not only physically interacts with KIR3DS05, its association also stabilizes cell surface expression of this stimulatory KIR protein.

### Stimulatory Signals of Activating KIR Are Transmitted via FcRγ

**Figure 5:**
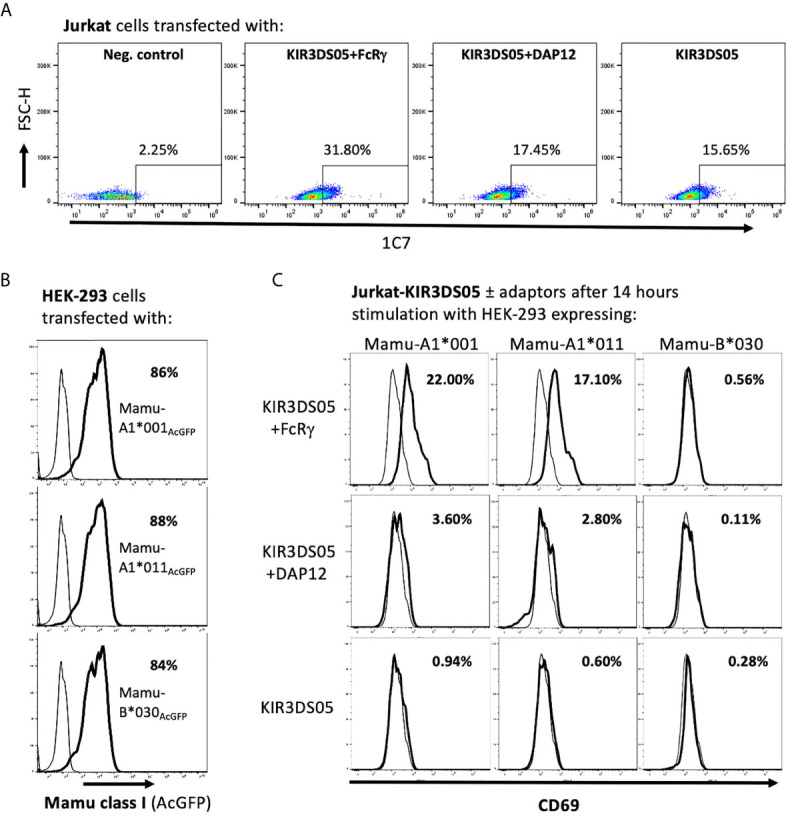
Signal transduction *via* adaptor protein resulting from interaction of KIR3DS05 with its cognate ligands. Transfected (bold lines) and untransfected control (thin lines) cells are shown. **(A)** Jurkat cells were transfected with plasmids carrying AcGFP-tagged KIR3DS05 and either myc-tagged DAP12 or FcRγ. Cell surface expression of KIR3DS05 was analyzed in FACS using antibody 1C7. **(B)** Flow cytometric analysis of AcGFP expression in HEK-293 cells stably transfected with constructs encoding AcGFp-tagged versions of Mamu class I that were described previously (25, 27). **(C)** The transfected Jurkat cells were incubated for 14 h with the HEK-293 cells expressing the KIR3DS05 ligands Mamu-A1*001 and A1*011 or the non-interacting Mamu-B*030 as negative control. Recognition of the ligands and signal transduction in the transfected Jurkat cells was monitored by CD69 expression in flow cytometry.

After demonstrating that FcRγ is the proper adaptor protein for activating KIR, we investigated whether signal transduction can be demonstrated in the presence of FcRγ as compared to DAP12. KIR3DS05 was particularly suitable as its ligand specificity was determined by us before (25). For this, we transfected KIR3DS05_AcGFP,_ KIR3DS05_AcGFP_+FcRγ_myc_ or KIR3DS05_AcGFP_+DAP12_myc_ in Jurkat cells. Also in Jurkat cells, which lack endogenous expression of both FcRγ and DAP12, the expression of KIR3DS05 was higher in the presence of FcRγ (31.8%) as compared to DAP12 (17.5%) or without adaptor protein (15.7%; **Figure 5A**). These transfected cells were then stimulated for 14 h with HEK-293 cells expressing KIR3DS05 ligands Mamu-A1*001_AcGFP_ and Mamu-A1*011_AcGFP_ as well as the non-interacting Mamu-B*030_AcGFP_ as control. The Mamu class I protein expression of about 85% positive cells and the MFI according to AcGFP expression was comparable between the three cell lines (**Figure 5B**). As readout of stimulation, we measured CD69 expression on Jurkat cells by flow cytometry. Following stimulation with the cognate ligands Mamu-A1*001 and Mamu-A1*011, CD69 is strongly induced (17.1-22.0%) on Jurkat cells expressing KIR3DS05+FcRγ. In contrast, very little CD69 expression was noticed on Jurkat cells expressing KIR3DS05_AcGFP_ alone (0.6-0.9%) or in combination with DAP12_myc_ (2.8-3.6%; **Figure 5C**). In the presence of the non-interacting Mamu-B*030, no CD69 induction was seen in all three experimental settings as anticipated (**Figure 5C**).

**Figure 6:**
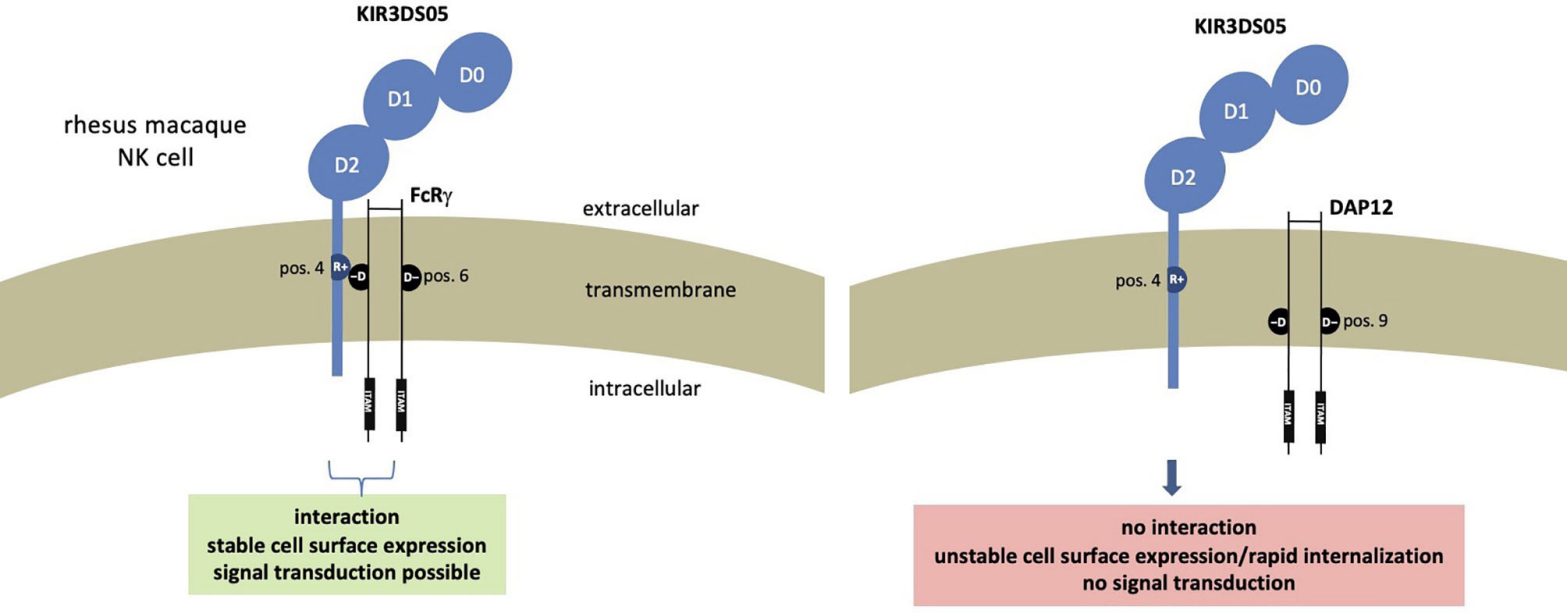
Functional consequences resulting from presence of different adaptor proteins in rhesus macaque NK cells expressing stimulatory KIR receptors.

Altogether these results demonstrate that in rhesus macaques the FcRγ protein is the adaptor that interacts with activating KIR such as KIR3DS05. The presence of FcRγ is necessary not only for the stable expression of KIR3DS05 on the cell surface, but also for signal transduction. **Figure 6** summarizes our findings and the functional consequences of differential presence of the two adaptor proteins for expression and function of activating KIR in rhesus macaques.

## Discussion

The association of signaling adaptor molecules and activating KIR proteins in Old World monkeys such as rhesus macaques was unknown so far. Here we could unambiguously show by different methods that a prototypical activating KIR protein of rhesus macaques associates with the FcRγ and not the DAP12 adaptor protein.

For the simultaneous expression of an activating KIR protein together with either the FcRγ or DAP12 adaptor, we used an IRES-containing expression vector. To ensure equal expression of FcRγ and DAP12 adaptor proteins, we placed the genes for adaptors upstream and the KIR3DS05-encoding gene downstream of the IRES sequence in the expression vector. The downstream position of a gene is known for lower expression efficiency as compared to the position upstream of the IRES (28). Through this order of the genes in the expression construct, we achieved not only simultaneous expression of adaptor and KIR, but also that the amount of KIR3DS05 is likely less than the adaptor protein and, hence, KIR3DS05 and not the adaptor protein would (if at all) be the limiting factor in the following experiments. After demonstrating the association of KIR3DS05 and FcRγ by co-immunoprecipitation in transfected HEK-293 cells as well as in rhesus macaque PBMCs, we analyzed in transfected cells the surface expression of KIR3DS05 in the presence or absence of adaptor proteins in flow cytometry. These experiments showed that the highest percentages of KIR3DS05-positive cells as well as the highest mean fluorescence intensity were achieved when FcRγ was present and not DAP12. Furthermore, also confocal microscopy not only indicated that KIR3DS05 co-localizes with FcRγ but showed also the highest expression level of KIR3DS05 in this combination. These data indicate that the KIR-interacting FcRγ obviously stabilized the cell surface expression of KIR3DS05 and that its absence results in low cell surface expression of KIR3DS05. Indeed, when we followed the heterologous expression of KIR3DS05 with or without adaptors over several cell passages, its expression remained high in the presence of FcRγ with just very few loss over time, whereas the expression of KIR3DS05 with DAP12 or without any adaptor protein rapidly decreased over time. Thus, the higher percentage of KIR3DS05-expressing cells that was noticed after transfection is most probably due to the stabilizing capability of FcRγ. This also indicates a crucial role of FcRγ in the regulation of stimulatory KIR3D proteins in macaque NK cells. Indeed, when FcRγ is absent or only the ‘wrong’ adaptor is present, signal transduction upon interaction with a cognate ligand is abrogated as shown by our co-incubation experiments with rhesus macaque MHC class I proteins. It should be mentioned that we noticed a small but statistically significant effect of the presence of DAP12 on the stability of cell surface expression of KIR3DS05 (**Figures 2** and **4**). A possible explanation for this observation might be that we used transfected cells with a strong promoter and, thus, higher levels of adaptor expression than under normal conditions of endogenous promoter control. Indeed, this effect was only seen shortly after transient transfection (**Figure 2**) or after start of a time-course kinetic experiment with stably transfected cells (**Figure 4**). This ectopic expression of DAP12 in the cell membrane may contribute to slightly stabilizing KIR3DS05 cell surface expression on the short term, but due to lack of molecular interaction, DAP12 is not able to stabilize it over time.

It was previously shown that genes encoding activating KIRs in rhesus macaques were most probably derived from recombination involving *KIR2DL4* and inhibitory *KIR3D* genes (14, 23). Thus, our data shown here are not only in accord with human KIR2DL4 associating with FcRγ (5), our data also support the finding that activating KIR in macaques were indeed derived from such gene recombination during evolution of Old World monkey *KIR* genes. Besides Old World monkeys, an arginine residue instead of a lysine in the transmembrane region of activating KIR is also present in New World monkeys (29, 30) and in small apes (31). In contrast, all hominid species (great apes and human) display a lysine residue in activating KIR proteins that associate with the DAP12 adaptor. This indicates that in the evolution of primates, a change from FcRγ to DAP12 in the usage of the activating KIR-associating adaptor protein along with corresponding adaptations in the transmembrane region of activating KIR were fixed in the lineage leading to extant hominid primates. Hence, the usage of FcRγ for activating KIR in platyrrhini (New World monkeys), catarrhini (Old World monkeys) and gibbons is likely the ancestral situation. The reason for the change in the usage of specific adaptors for activating KIR in hominid primates is unknown. The evolution of KIR genes in primates is very dynamic and frequently leads to formation of new genes by recombination (14–23), most probably driven by infections. Thus, it appears advantageous to have at hand different opportunities for adaptor proteins in order to cope with different requirements from activating KIR. Indeed, the broad expression of FcRγ, DAP12 or DAP10 in different cells of the immune system, their association with diverse receptors and their stabilizing role of receptor expression mirror their importance and their regulatory roles (10, 13, 32, 33). Adaptive NK cells in humans generated as a consequence of infection with cytomegalovirus (CMV) are characterized by loss of FcRγ expression through epigenetic silencing (34, 35). While such loss of FcRγ is thought to result in concomitant loss of stimulatory receptors as shown for NKp46 and NKp30 (8, 35–38), it also results in increased killing capacity of adaptive NK cells mediated *via* CD16 (34, 35, 39). This might be explained by association of CD16 with both FcRγ and TCRϛ signaling proteins (7, 40–42) and the higher number of ITAMs in TCRϛ compared to FcRγ. Thus, CMV-associated adaptive human NK cells are regulated by loss of FcRγ to sharpen CD16-mediated recognition of antibody-tagged cell targets. FcRγ-deficient adaptive NK cells were also reported in CMV-infected rhesus macaques (43). We hypothesize, that loss of FcRγ in adaptive NK cells of macaques not only sharpens their CD16-mediated effector function as described previously by others (44), but that concomitant loss of activating KIRs further refines their immune function: moving towards antibody-driven and away from ligand (MHC class I)-driven recognition. If this would indeed be the case, then macaque activating KIRs are expected to have a more prominent role in shaping adaptive NK cell functions as their human counterparts.

## Data Availability Statement

The original contributions presented in the study are included in the article/supplementary material. Further inquiries can be directed to the corresponding author.

## Author Contributions

LW conceived the project. MZH and LW designed the experiments and wrote the manuscript. MH carried out all experiments. MH and LW analysed the data. All authors contributed to the article and approved the submitted version.

## Conflict of Interest

The authors declare that the research was conducted in the absence of any commercial or financial relationships that could be construed as a potential conflict of interest.
